# Transforming Patient Experience: Health Web Science Meets Medicine 2.0

**DOI:** 10.2196/med20.3128

**Published:** 2014-03-20

**Authors:** Lynn-Sayers McHattie, Grant Cumming, Tara French

**Affiliations:** ^1^The Institute of Design InnovationThe Glasgow School of ArtGlasgowUnited Kingdom; ^2^University of Highlands and Islands and University of AberdeenAberdeenUnited Kingdom; ^3^University of the Highlands and Islands, Moray CollegeElginUnited Kingdom

**Keywords:** patient-centered medicine, co-creation, co-design, Health Web Science, Medicine 2.0, P4 medicine

## Abstract

Until recently, the Western biomedical paradigm has been effective in delivering health care, however this model is not positioned to tackle complex societal challenges or solve the current problems facing health care and delivery. The future of medicine requires a shift to a patient-centric model and in so doing the Internet has a significant role to play. The disciplines of Health Web Science and Medicine 2.0 are pivotal to this approach. This viewpoint paper argues that these disciplines, together with the field of design, can tackle these challenges. Drawing together ideas from design practice and research, complexity theory, and participatory action research we depict design as an approach that is fundamentally social and linked to concepts of person-centered care. We discuss the role of design, specifically co-design, in understanding the social, psychological, and behavioral dimensions of illness and the implications for the design of future care towards transforming the patient experience. This paper builds on the presentations and subsequent interdisciplinary dialogue that developed from the panel session "Transforming Patient Experience: Health Web Science Meets Web 2.0" at the 2013 Medicine 2.0 conference in London.

## Introduction

### The Imperative of Change

There is a consensus that the current modes of health care delivery are unsustainable [1,2]. For more than a century, the successful and dominant model in controlling infectious diseases in Western medicine has been biomedical in nature and underpinned by controlled clinical trials [3]. This model has increasing limitations within its paradigm for the social, psychological, and behavioral dimensions of illness [4]. The predominance of the biomedical model is now being challenged. Infectious diseases, the challenge of the 19th and 20th centuries, have given way to the prevalence of chronic disease [1,5]. These chronic conditions are closely related to lifestyle choices that arguably account for 55% of deaths of people aged 15 to 64. This contrasts with statistics from a century ago, where 5% of deaths were attributable to personal decisions, while infectious diseases accounted for most of the deaths [6]. In response, medicine is beginning to embrace the biopsychosocial model, emphasizing patient-centered care delivered by interdisciplinary provider teams [7]. This biopsychosocial model is a call to change our way of understanding the patient and to expand the domain of medical knowledge to address the needs of each patient [8]. The future of health care in this era of chronic disease requires increasing effort directed towards improving personal choices regarding life risks [6] and requires the full engagement of people in their own health care and lifestyle decisions [5,9,10].

This viewpoint paper argues for a new approach to understand behaviors and motivations, which involves individuals and their communities, and critically addresses the socioeconomic divisions that continue to underpin and determine lifestyle choices [11]. Design approaches can contribute to addressing the important complexities and challenges in the current health care model and in so doing develop innovative approaches in the application of digital, Web-enabled, and mobile technologies for future care.

### The Role of Information and Communication Technologies

In the 1990s, health information and communication technologies (ICT) offered promise to mitigate the problems facing the delivery of health care [12]. However, it required the cultural shifts that social media and mobile devices have catalyzed in recent times to align with the recognition that many health care systems are now at a tipping point [1]. New approaches are thus required to galvanize communities working in ICT and health to integrate the Internet and related technologies in the delivery of person-centered health care [12]. Internet-delivered interventions have the potential to combine the tailored approach of individual or face-to-face interventions, while maintaining the scalability of public health interventions with low marginal costs per additional user. It is incumbent on those developing health technologies delivered via the Internet to embrace new methodologies in design and evaluation and recognize the limitations of those already utilized [13].

### Digital Health Care, Personalized Medicine, and Digital P4 Medicine

The future of medicine is increasingly mediated through preventative, participatory, personalized, and predictive modes known as P4. Digital P4 Medicine broadly defines personalized medicine as a health care paradigm that uses a range of technologies from the fields of ICT, medical equipment, and pharmaceutical devices to deliver P4 medicine [14]. In 2003, Leroy Hood introduced the term P4, with the vision that it would transform the practice of medicine, moving it from a largely reactive discipline with an emphasis on sickness and treatment to a proactive one [15]. Under this model, patients are expected to benefit from better diagnoses leading to individually targeted and thus more effective treatments as a consequence of the new forms of active participation by patients in the collection of personal health data with the recognition that this approach requires a shift to a patient-centric model.

**Figure 1:**
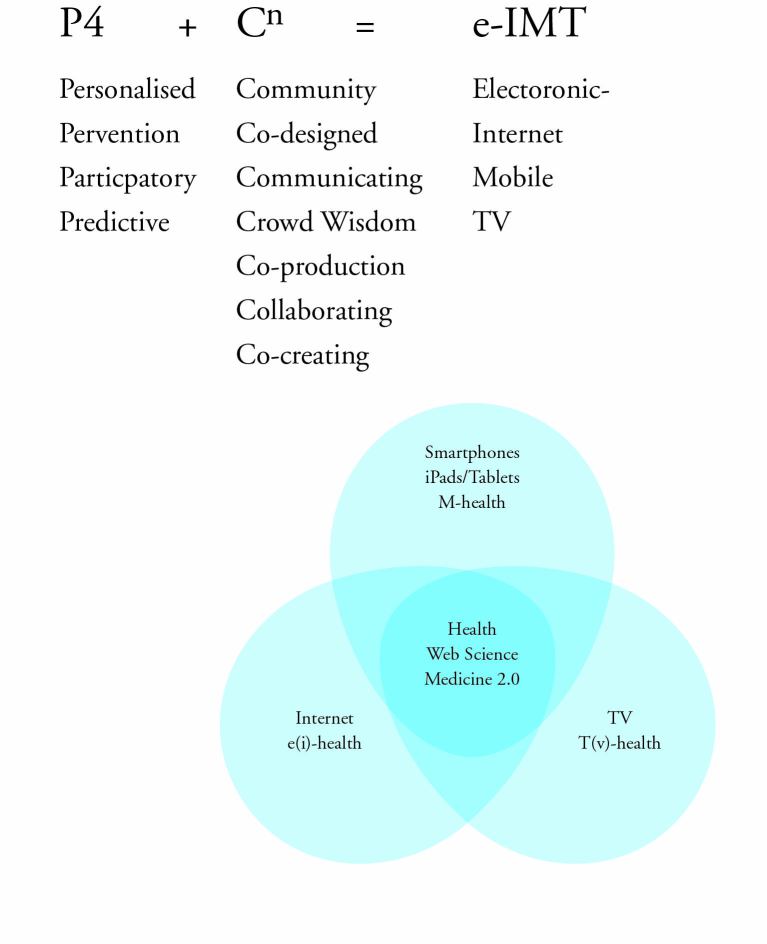
The preventative, participatory, personalized, and predictive (P4) model.

Figure 1 illustrates the digital health ecosystem that is being developed in the North of Scotland with its ethos on co-design and collaboration underpinned by the disciplines of Health Web Science (HWS) and Medicine 2.0 to mitigate against the development of fixed milestones and rigid methodologies of previous eHealth innovation [16].

### Health Web Science and Medicine 2.0

Owing to current models of health care being unsustainable, new digital eHealth frameworks such as P4 + Cn= eIMT (Figure 1), are needed and new approaches informed by design, incentivization, and evaluation. HWS, a sub-discipline of Web Science [17,18], studies the interaction of health and the Web, and as such complements disciplines that come under the umbrella of Medicine 2.0. The focus of HWS requires an understanding of networks and is therefore more strongly aligned with non-medical stakeholders than Medicine 2.0 [19]. The disciplines of and related to HWS and Medicine 2.0 therefore have potential to provide frameworks and leadership in integrating eHealth into mainstream health care delivery. New approaches are therefore required to understand the complexities and enable design and co-design of innovative approaches using digital and Web-enabled technologies in health care. Underpinning the paradigm shift from a treatment model to self-caring medicine relies on collaborative principles combined with an agile methodology across multiple platforms thus ensuring engagement with the target audience.

Design interventions provide a framework for the marriage of Health Web Science and Medicine 2.0, specifically investigating how technology can support digital health interventions. Design interventions present enormous opportunities underpinned by behavioral science [20], to leverage the potential of exponentially growing innovation into an integrated framework that provides personalized health care.

### Design-Led Approaches for Person-Centered Care

Drawing together ideas from design practice and research, complexity theory, and participatory action research (PAR), we discuss design as an approach that underpins concepts of person-centered care. From complexity theory, we are interested in how modes of interaction and connection, combined with non-linear processes, can give rise to innovation, particularly in the digital domain. Participatory action research is increasingly being utilized as a methodology from a patient-centered perspective. In connecting these, we are aware that design-led approaches are fundamentally social and linked to concepts of person-centered care. Since the turn of the 21st century, health care researchers have begun to apply complexity theory [21], including the theory of complex adaptive systems. Complexity theory describes systems that are capable of spontaneously reconfiguring themselves through the repeated application of simple, order generating rules in a process known as self-organization [22-24]. Non-linearity, interconnectedness, and positive feedback loops are key concepts in understanding the nature of these self-organizing processes. While complexity theory has helped develop alternatives to mechanistic approaches and focuses on creativity, it could be argued that it provides little insight into the nature and role of individual and participatory action in the context of person-centered care.

Participatory action research has special resilience and value in this emerging field of inquiry. PAR is grounded in the participative, interdependent ecosystems of social life. It builds feedback loops into ongoing research and can be used for monitoring complex adaptive systems. PAR brings together action and reflection, theory, and practice, in the pursuit of solutions that link practice, ideas, and innovation towards the human flourishing of individuals and collectively as communities [21]. It is an orientation to inquiry that seeks to create participative communities of practice and communicative spaces around key focal issues. Typically, these communities are interdisciplinary, require multiple perspectives, and engage in a process of action and reflection whereby the cycles of action and reflection integrate multiple ways of knowing and doing.

PAR is rooted in participation; it has ushered in human interaction while focusing attention away from notions of a system in which research is done to people and towards a view of individual and collective participation. PAR is a methodology based on reflection, data collection, and action.  It aims to improve health and reduce health inequities by involving the people who, in turn, will be motivated to take actions to improve their own health [25]. Cooperative inquiry comes under the umbrella of PAR [26].  The aim of cooperative inquiry is to research with rather than on people. It emphasizes that all active participants are fully involved in research decisions. These approaches lend themselves well to an agile methodology, whereby each iteration or cycle of development is evaluated and the lessons learned then fed into the next cycle.

### Collaborative Design

Collaborative design is conducted in collaboration by a coalition of researchers and practitioners, community members, patients, health professionals, and other stakeholders. The research inquiry includes three elements: systematic inquiry, design practice and design interventions. Through drawing together ideas from design practice and research, complexity theory, and participatory action research, we are establishing a link between social processes and participation that underpin concepts of person-centered care.

The terms participatory design, co-production, co-creation, and user-centered design, amongst others, are used in design literature. Sanders and Stappers [27] referred to co-creation as any act of collective creativity; creativity that is shared by two or more people. Co-creation is a generic term with applications ranging from the physical to the metaphysical and from the material to the spiritual. Sanders and Stappers [27] defined collaborative design or co-design, as collective creativity applied across the whole span of the design process. Thus, co-design is a specific instance of co-creation. The term co-design refers to actants being actively involved in interdisciplinary networks and participatory action to foster unique partnerships, products, or processes. Design methodologies provide a flexible framework that, consistent with complexity theory, are cognizant of the indeterminate nature of the social situation and its inherent unpredictability. Design innovation is an inclusive and iterative process that utilizes design methods and collaborates with people to develop and prototype innovative ideas that lead to sustainable solutions and valuable outcomes.  Design innovation as a collaborative approach views research as a set of experimental and emergent practices that can broaden the ways we understand social processes and behavior. It utilizes an agile, action-orientated methodology, and direct engagement with people and their experiences in relation to focal issues. The rich mix of personal, sociocultural, and contextual influences, provide the basis for documenting and producing visual schema as a means of communication. It is this precise relationship between participation, research and design that can reveal deep insights. Having outlined our theoretical position, the role of design innovation, and co-design, we now present a design research approach entitled “cube”.

## The Cube Research Approach

The cube research approach involves three people working together in stages of three days, for three times, that is, three cubed [28].  The cube is an agile method of design research within a thematic territory. A cube is an intervention that is designed to create trajectories or tangible outcomes around a focal issue, while allowing for an open approach to the research process. An interdisciplinary team of three people including a design researcher, a design practitioner, and a subject specialist undertake a project within a defined research theme and work for nine days each, totaling 27 person-days, to deliver trajectories, propositions, or solutions. The rhythm of the work is self-organized by the cube participants along with the organization of the project, the roles and methods.

**Figure 2:**
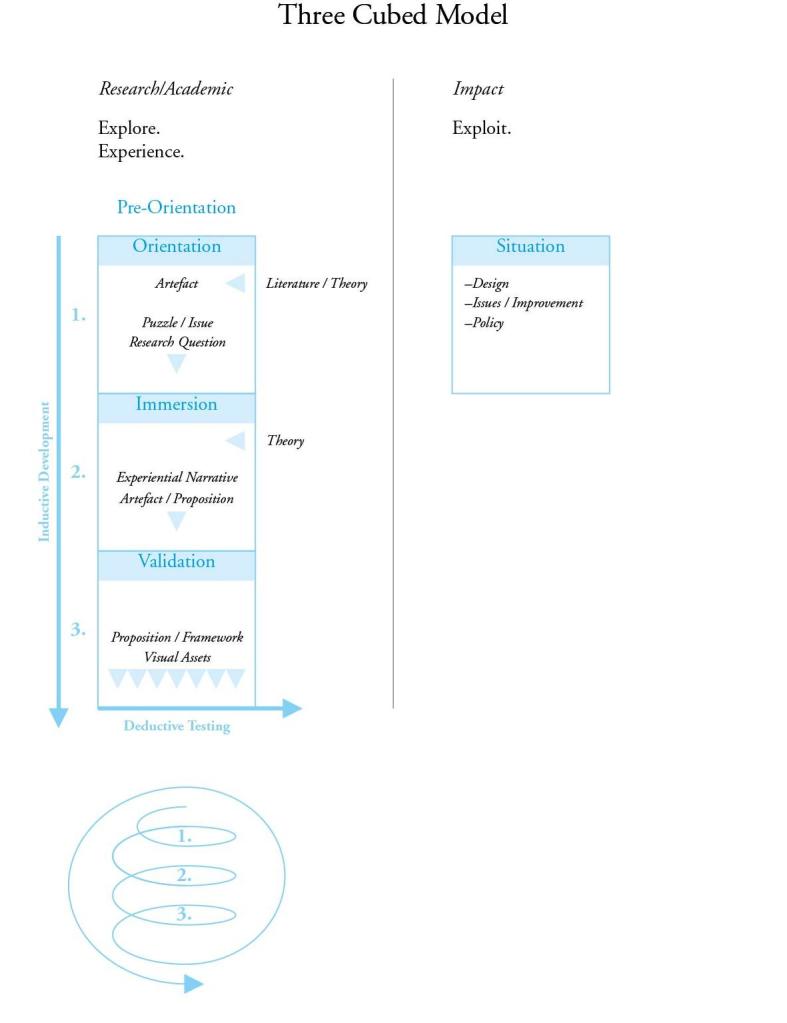
The Three Cubed Model.

Figure 2, illustrates the two different types of cube contribution (ie, academic or impact) and the expected iteration of each. Following a pre-orientation phase where the team is focused on the thematic territory, the cubes will undergo up to three iterations of inductive development. In the first iteration—orientation—design tools or visual artifacts will be created to explore the thematic issue and the research team would define the research approach. In the second iteration—immersion—the researchers would immerse themselves within the context of the inquiry and seek to develop participatory and experiential narratives and if applicable, introduce or make artifacts to interweave these visual and verbal narratives. Finally, in the third iteration—validation—they would seek to validate earlier findings and produce high quality visual assets towards deductive testing.

The purpose of a cube is to address research themes with diverse teams of collaborators and expertise working together for short periods. The background knowledge of the researchers and participants is applied and developed quickly within a fast-paced collaborative space. The cube approach is designed to contribute to both academic debates and deliver impact at a wider societal level. The research approach is focused on the levels of collectivity required between the individual and the community aligned to specific focal issues and societal challenges. Design approaches have been developed to establish empathy between practitioners, researchers, collaborators, and participants in the context of health and care. This approach aims to develop designers’ multi-sensory and non-verbal understandings of complex health, care, and wellbeing from an otherwise inaccessible perspective towards a richer comprehension of inclusive design for a diverse population. Design practice and multisensory comprehension suggests an aesthetic approach through which designers can build empathic, intuitive, and productive relationships with patients, participants, and collaborators.

## Health Ecosystem

This research approach requires the participation of all the parties involved in the delivery of the health care being studied. This grouping of parties can be described in terms of a health ecosystem or network. This investigation of networks prioritizes links between innovation and creative capital that in turn determine the ways in which apparently disparate resources—physical, social, and material—can be usefully related to create communities within an ecology of social and cultural care.

Design approaches expand the vision of an ecology of social and cultural care with the ability to diffuse new approaches in the future of work and organizations, which are required to develop a healthy ecosystem that will in turn innovate future models of care. The organizations involved in health care delivery form a complex social ecosystem with demand being met from a variety of formal and informal sources.  By nature these ecosystems are made up of diverse and varied groups that interact within the constraints set by the changing environment.  In the current model, incorporation and implementation of new methods and innovations can take upwards of ten years [29] and are initiated and implemented by large health care providers in most instances.  PAR lies within the quality of interaction and the way in which we work towards a view that enables health care providers to develop an ecosystem which also encompasses and includes those receiving care and the communities which support them. Design approaches facilitate the inclusion of these groups into the ecosystem through the development of new methods, new tools, and new partnerships.

These networks, partnerships, and collaborations extend to involving ministerial leadership, life sciences, enterprise, academia, health and social care, and co-designing involvement from the public towards developing personal ownership in behavioral change.  These networks which may challenge conventional delivery models, enabled by digital technology, can then lead to the accelerated adoption of new ways of working for health care providers and innovative modes of self-care for citizens aligned to the advancing role of technology in personal care paradigms.

## Discussion

The role of technology is critical as a conduit in the development of participatory platforms. Within a dynamic digital age, the understanding and implementation of design systems and innovative networks can create person-centered care experiences and services, which are relevant to target audiences and markets. Deep insights into the needs of people and the imagination of end-users are vital for creating new design-led digital solutions and experiences in understanding the social, psychological, and behavioral dimensions of illness and the implications of transformational change.

Our theoretical positioning, the role of design and the depiction of co-design as participatory action in Health Web Science and Medicine 2.0 contexts has led to a number of early impressions at this stage in our inquiry. Participatory action research is increasingly being utilized as a methodology from a patient-centered perspective. Indeed some have proposed that participative approaches and co-design are fundamental to the personalization and the digital transformation of all public services [30]. The aim being to make recommendations for good practice that will tackle a problem or enhance the performance of the organization and individuals through changes to the community within which they operate [31]. In particular we are concerned with designing participatory research approaches, which are emergent and experimental [21]. We are interested in the role of design research and practice and specifically co-design in understanding the social, psychological and behavioral dimensions of long term conditions and the implications for the design of future care. In so doing we are questioning some of the traditions of the Western biomedical paradigm geared towards known outcomes and engaging in designing innovative approaches towards sustainable solutions.

In some ways, we are working with the idea of design research and practice as a participatory framework of social-material interactions. We are proposing a contemporary approach to design research that has moved from the design of products to design which is embedded in the understanding of social processes through developing networks of extreme expertise and collaborations between design researchers and practitioners, health professionals, clinicians, patients, and other stakeholders around substantive issues that in turn will transform the patient experience.

## Abbreviations

HWS: Health Web Science
ICT: information and communication technologies
P4: predictive, personalized, participatory and preventative
PAR: participatory action research
